# KnetMaps: a BioJS component to visualize biological knowledge networks

**DOI:** 10.12688/f1000research.16605.1

**Published:** 2018-10-17

**Authors:** Ajit Singh, Christopher J. Rawlings, Keywan Hassani-Pak

**Affiliations:** 1 Computational and Analytical Sciences, Rothamsted Research, Harpenden, AL5 2JQ, UK

**Keywords:** knowledge network, knowledge graph, network visualisation, knowledge discovery, biojs

## Abstract

KnetMaps is a BioJS component for the interactive visualization of biological knowledge networks. It is well suited for applications that need to visualise complementary, connected and content-rich data in a single view in order to help users to traverse pathways linking entities of interest, for example to go from genotype to phenotype. KnetMaps loads data in JSON format, visualizes the structure and content of knowledge networks using lightweight JavaScript libraries, and supports interactive touch gestures. KnetMaps uses effective visualization techniques to prevent information overload and to allow researchers to progressively build their knowledge.

## Introduction

Networks have been widely used to visually represent complex information in many disciplines, ranging from social sciences ( Szell *et al.*, 2010) to engineering, physics, biology, computer science, design and manufacturing ( Wang & Alexander, 2015). They fulfil the need to present a system, not only as individual entities but as a whole, by capturing the myriad inter-linked components within the system ( Pavlopoulos *et al.*, 2011). Networks are represented as graphs comprising a set of nodes connected by edges. Networks can be homogeneous with all the nodes within the network being of the same type, or heterogeneous with nodes and edges of various types ( Sun & Han, 2012). Recently, the term knowledge network or graph has been used frequently in research and business, usually in close association with Semantic Web technologies and linked data. Knowledge networks are increasingly used to model diverse knowledge domains by acquiring and integrating information into an ontology and applying a reasoner to derive new knowledge ( Ehrlinger & Wöß, 2016).

A challenge when visualizing knowledge networks is to avoid information congestion and overload that could hinder user experience. The potential richness of data captured in the attributes and density of connections makes it a greater challenge to use standard network visualization tools which often focus on simply visualizing the structure of the network itself ( Becker *et al.*, 1995). In molecular biology, there is a wealth of available information, and visualizing all of it at once reduces the value of a visualization or makes it even unusable for analytical purposes, and therefore requires the development of special approaches when visualizing such data ( Vehlow *et al.*, 2015).

Previously, our group developed a web-based tool, Ondex Web ( Taubert *et al.*, 2014), for visualising knowledge networks generated with the Ondex data integration platform ( Kohler *et al.*, 2006). It supported the Ondex exchange language (OXL) and was predominantly used to visualise a biological knowledge domain. However, being a Java-applet and using legacy web technologies, it constantly led to compliance concerns on different web browsers, which hindered its reusability. The advance of modern JavaScript-based data visualisation libraries such as cytoscape.js ( Franz *et al.*, 2016) and jQuery ( Benedetti & Cranley, 2011) has made it possible for us to learn from our experience with Ondex Web and to develop a new lightweight and reusable component optimised for the visualisation of content-rich knowledge networks.

In this paper we describe KnetMaps ( Singh & Hassani-Pak, 2018), an interactive BioJS component to visualise integrated knowledge networks. It is well-suited for applications that require scientists to visualise complementary types of evidence in a single interactive view. KnetMaps is an important visualisation component of KnetMiner where it is used for visualising knowledge networks of crop genomes ( Hassani-Pak *et al.*, 2016) and supporting scientists to make informed decisions in gene and trait discovery research. It uses a generic design and hence can be readily embedded in other knowledge discovery applications.

## Methods

**Figure 1.**
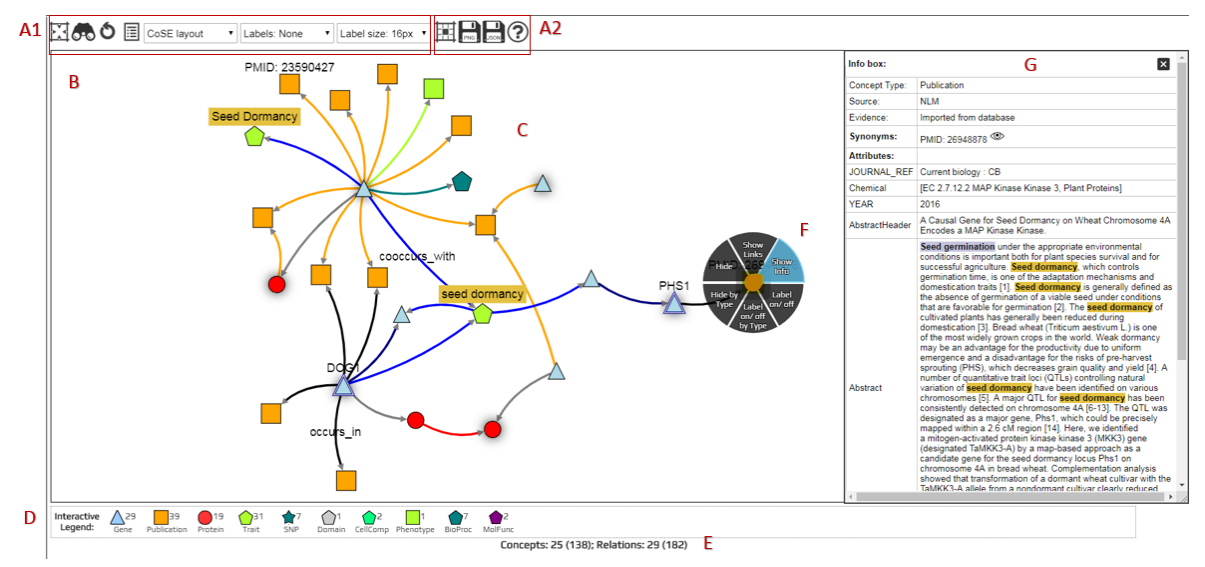
The main components of KnetMaps. ( **A1**) Display configuration (set network layout, node/edge visibility, label visibility and size). ( **A2**). Network export options (cytoscape JSON, PNG). ( **B**) Interactive network container (pan and zoom the network), ( **C**) Knowledge network rendering (displays nodes and edges of different types). ( **D**) Interactive legend (to overlay more linked entities to the visible network). ( **E**) Network summary statistics (indicates hidden and total nodes/edges). ( **F**) Touch-sensitive context menu (show further information about selected node/edge, hide selected entity or its label, show hidden linked entities). ( **G**) Item Information panel (display content-rich attributes of selected node/edge).

The KnetMaps component has been developed as part of the KnetMiner software suite and follows the standards set by the BioJS registry. KnetMaps employs a variety of network visualization techniques such as interactive controls, information juxtaposition and data filters. Using effective visualization techniques it prevents information overload and allows researchers to progressively explore and reveal the inter-connected entities within the larger knowledge network ( Figure 1).

### Visualising knowledge networks

Visualisation of knowledge networks needs to consider two key criteria: i) the heterogeneous and interconnected nature of the network and ii) the content-rich attributes of nodes and edges that cannot be easily displayed as part of the actual network.

Nodes in a biological knowledge network represent entities such as genes, proteins, phenotypes, pathways, publications and ontology terms; connected by edges of various types such as “encodes”, “published_in” and “ortholog” ( Figure 1C). KnetMaps visualises each node type using a customized combination of shape and colour. Edge types are rendered using a combination of distinct size and colour attributes. The position of nodes and the length of edges is calculated using a force-directed layout that enables connectedness, separation and pattern-based clustering of closely inter-linked entities ( Dogrusoz *et al.*, 2009). Labels can be added to nodes and/or edges to enable easier understanding of the underlying data.

To view the potentially rich set of key-value attributes on nodes and edges, we have developed the Item Information panel ( Figure 1G). It displays all textual (e.g. abstract, title of a publication) and numeric properties (e.g. accessions, scores and weights) of a selected node or edge in table format, including annotations, detailed descriptions, secondary labels and links to external websites and databases about the selected entity. Users can also use the information displayed in this panel to customize the rendered visualization of node and edge labels to their needs.

### Intuitive user interaction

On relevant devices, KnetMaps supports basic touch gestures such as tap, tap-hold, tap-drag and pinch and zoom for user interaction. Users can interact with individual nodes and edges in the rendered network by using standard mouse or touch gestures such as click or tap gesture on a specific node or edge to get a summary of its properties or use the mouse wheel or pinch gesture to manipulate the zoom settings on the network.

Users can right-click or tap-hold on a node or edge to activate a radial context menu ( Figure 1F) that provides a range of easy-to-use mechanisms for exploring or manipulating the selected entity. Users can click or tap on a node or edge and view further information such as type, description and annotations, summarised in a dialog box. Users can also tap-drag individual nodes or edges to re-align them within the visible network or tap-hold and reposition the rendered network as a whole.

The visualized network can be further explored and exported ( Figure 1A1) using a variety of menu functions. For example, networks can be exported from KnetMaps as images (in png format) or as cytoscape-compatible JSON which can then be opened in the Cytoscape desktop application ( Kohl *et al.*, 2011) for further downstream analysis.

### Incremental approach to exploratory analysis

KnetMaps controls information overload in the visible network by providing means to overlay data and extend it in incremental steps, thereby adopting a progressive approach where a subnetwork of interest from the underlying knowledge network is initially visualized and end-users are given the means to add more related information to the visible network. The subnetwork of interest is determined by the application using KnetMaps and passed to it through a “display” attribute in the API/JSON. KnetMaps generates a summary of the number of visible/hidden entities in the knowledge network so that end-users have an overview of what information might be present in the knowledge network but is currently hidden in the visible network. This information is automatically updated each time the user reveals or hides entities from the visible network ( Figure 1E).

The first way of adding additional information to the network is by using the *interactive legend* that gives a summary of all node types present in the network, along with a numerical count of the total number of nodes of each type ( Figure 1D). For example, clicking on a “Publication” symbol in the legend will add publications linked to visible gene and protein nodes, thereby enabling users to expand the visible knowledge network in real-time.

The second way to add or hide information is by using the *context menu* ( Figure 1F). It allows users to hide individual nodes and edges, or hide all nodes and edges of a particular type. This can be useful for removing irrelevant or noisy information from the visible network. Additionally, it allows in- and out-going relations to be added to a selected node when these were initially hidden. This can be useful when a node acts as a knowledge hub, but only a small subset is initially visualized to intentionally prevent information overload, or if a node is part of a larger, more intricate knowledge pathway. In such cases, users can rapidly overlay connected entities within the selected node’s neighbourhood onto the visible network to effectively connect the dots and explore the myriad relationships between the network entities.

## Implementation

KnetMaps leverages CytoscapeJS v.2.4.7 and jQuery v1.11.2 to visualise knowledge networks. It has been designed in a modular fashion and made available in NPM and BioJS, making it a reusable plug-and-play component within dynamic web applications.

### Input data model

KnetMaps loads JSON input data (streamed or locally stored) and renders it as a knowledge network. It uses the cytoscapeJS JSON format specification in which the network is modeled as nested “nodes” and “edges” array objects. Each node or edge entity has a set of required properties such as colour, shape, size, identifier, label, border and visibility. We have extended the cytoscapeJS schema with an additional JSON object to store optional node/edge properties, e.g. abstract and title of a “Publication” node. The separation of required visual properties and optional data specific information, provided a more efficient way of rendering the general network while displaying node/edge specific information on demand, e.g. when the user clicks on a node.

### Network rendering

Networks are rendered using a cytoscapeJS-based network stylesheet that maps the set of required JSON properties to the network object. The KnetMaps generator stylesheet sets the shape and colour of a node based on parameters provided for it in the JSON input dataset, e.g., ‘shape: data(conceptShape)’ and ‘background-color: data(conceptColor)’ where ‘conceptShape’ and ‘conceptColor’ are properties with set values in the input dataset. Developers can customize the stylesheet to replace the supported static cytoscapeJS shapes (such as triangle, round-rectangle, ellipse, pentagon and star) with images. CytoscapeJS selectors have been incorporated in the network stylesheet to filter nodes and edges based on these interactions and add functions that toggle their visual attributes such as highlighting a node or edge when selected and toggling visibility of labels accordingly.

### Interactive knowledge display

KnetMaps provides various features for interactive and incremental data exploration by incorporating useful Javascript libraries, such as the cytoscape.js-cxtmenu widget and various force-directed layout libraries to render the knowledge network, including the CoSE layout ( Dogrusoz *et al.*, 2009), which is the default network layout used in KnetMaps. Other layouts that can be used by end-users include the physics-based force layout, the CoSE-Bilkent layout that provides additional network topology and geometrical constraints, or static in-built cytoscapeJS layouts, such as the pattern-based circular layout or the concentric layout. KnetMaps packages cytoscapeJS-compatible extensions to these layouts within the application distribution and incorporates optimised settings for each layout within the application itself.

### Scalability and performance

Networks of up to 1000 nodes and up to 3000 edges can be visualized in KnetMaps without significant performance degradation or visual delay in layout animations. Visualizing much larger networks (i.e. networks with over 10,000 nodes) increases the initialisation time and can cause jerky or delayed layout animation effects. Some of the rich visual styles used by KnetMaps can be somewhat expensive to render by cytoscapeJS, for example, rendering bezier curved edges.

The KnetMaps code addresses this by providing developers with flexible options to reduce the rendering complexity of the networks. All visual display settings have been made fully customizable to allow developers to tweak element styles such as node shape, edge curve and node border. Network container settings such as pixel ratio and motion blur can also be similarly easily altered, as can layout parameters such as reducing animation time, decreasing the number of layout iterations to run and disabling animation when rendering very large networks. The default parameters and settings work well in KnetMiner, based on the average sizes of the biological networks (between 300–1000 nodes) that it visualizes. However, customizing these parameters to employ simpler visual settings for larger networks can mitigate performance degradation during rendering.

### Operation

KnetMaps has been published to NPM and the BioJS ( Gómez *et al.*, 2013) registry, which provides a centralized portal of JavaScript tools and widgets used to analyse and visualize biological data, making it easy for research software users to install KnetMaps and embed within the HTML of their own web pages. The minimum system requirement is a PC with *npm* (part of *Node.js*) installed, a modern web browser with JavaScript enabled and a JSON sample file (see knetmaps/sampleFiles). Once *bower* for managing front-end components and *gulp* for bundling and distribution have been installed to the system ( *npm install bower gulp*), following steps are needed to install and bundle KnetMaps:

npm install knetmaps
cd knetmaps/
gulp optimise

The process will take a few minutes and create the *dist/* folder, containing further *img/*, *js/*, and *css/* subfolders which need to be copied to a web page’s root directory. Now, a simple *index.html* can be created to load and visualise the sample JSON dataset:

<head>
    <link href="css/knetmaps.css" rel="stylesheet" /> 
    <script src="js/knetmaps-lib.min.js"></script> 
    <script src="js/knetmaps.min.js"></script>    
    <script src="sample.json"></script> 
    <title>KnetMaps.js demo</title>
</head>

<body>
    <div id="knet-maps"/>
    <script type="text/javascript">KNETMAPS.KnetMaps().draw('#knet-maps');</script>
</body>

## Use cases

KnetMaps is used as a network visualization component within tools and platforms that visualize biological knowledge as an interactive network, such as the KnetMiner ( Hassani-Pak, 2017) and Daisychain. In KnetMiner, there is a need to visualise query related subsets of a genome-scale knowledge network ( Hassani-Pak *et al.*, 2016) in the web-browser. KnetMaps is one of the key components in KnetMiner to visualize and explore integrated information of inter-linked biological entities and processes to help in hypothesis generation and validation, and to accelerate candidate gene discovery. KnetMaps is also part of the KnetMiner Web API and therefore enables collaborators to view knowledge networks for specific genes and keywords from their own applications.

Daisychain is a web application that links genome annotations, aiming to enable researchers working on certain species genes to investigate homologs in other published assemblies via a web interface called Daisychain-Web. The application can be queried using keywords or FASTA sequences with statistical cut-offs and the search results, i.e., links between genes and annotations across similar or identical species or cultivars are visualized as a network using KnetMaps. Daisychain uses KnetMaps out-of-the-box for rendering and visualization and adds further annotation and filtering options to the Item Information panel.

## Conclusion

Visualizations are a useful mechanism employed in many disciplines to present information in an intuitive representation that enhances user cognition and helps identify unique patterns and important trends in data. Network formalisms are becoming an increasingly popular means to combine data from inter-connected sources into a concise representation for easier and intuitive exploratory analysis. KnetMaps has been implemented as a fast and lightweight touch-friendly tool for visualizing content-rich, heterogeneous knowledge networks. The implementation uses cytoscapeJS, jQuery and JavaScript extensions for interactive functionality to ensure that low-memory, touch-compatible networks can be rendered in web browsers without the need to write extensive and unwieldy server-side code. Usage of JavaScript ensures rendering compatibility with most web browsers without the need to install any additional software, e.g., Java Applet or Adobe Flash. KnetMaps provides an interactive means to display, filter and overlay networked knowledge, and visually traverse the relationships connecting information within the rendered network. It incorporates a host of visualization techniques such as juxtaposition and superposition to encourage a step-by-step exploration of larger volumes of disparate data, thereby enabling end-users to investigate and analyse inter-linked knowledge in an incremental and intuitive manner.

## Data availability

All data underlying the results are available as part of the article and no additional source data are required.

## Software availability

**Latest source code available from:** https://github.com/Rothamsted/knetmaps.js.

**Packaged distribution at BioJS:** http://biojs.net/#/component/knetmaps.

**Packaged distribution at NPM:** https://www.npmjs.com/package/knetmaps.

**Archived source code as at the time of publication:** https://zenodo.org/record/1434144 ( Singh & Hassani-Pak, 2018).

**Example demonstration of KnetMaps:** http://knetminer.rothamsted.ac.uk/KnetMaps/.

**License:** GNU General Public License v3.0.
