# Supplementary material for: KnetMaps: a BioJS component to visualize biological knowledge networks
Source: F1000Res. 2018 Oct 17;7:1651. [Version 1] doi: 10.12688/f1000research.16605.1 (PMC6347035; doi:10.12688/f1000research.16605.1)
Supplement: Peer review history

# Peer review: Referee response for version 1

**DOI:** 10.5256/f1000research.18148.r42475

**Published:** 2019-01-29

**Authors:** Ramil Mauleon

**Affiliations:** 1 Strategic Innovation Platform, International Rice Research Institute, Manila, Philippines

The functionality provided by KnetMaps is very useful for molecular geneticists (and the bioinformaticians who support them), whose aim is to identify the best candidate genes to use in trait improvement (I am coming from the bioinformatician/geneticist side). Using heterogenous evidences (omics experiment results, literature, function by association inference from sequence identity, GWAS results) is a common exercise in order to get a more informed list of candidate genes to work with. This is the strength of the visualization of Knetmaps: the ability to display the evidences incrementally, and to visualize the connection of evidences into an intuitive network display, which is very helpful for biologists in order to make decisions on which genes to select for further (expensive) experimental validation. Network rendering and response is fast, thus providing an enjoyable end user experience.

 I do share one reviewer's feedback on how to revert the network view back to the original state prior to clicking on an icon in the interactive legend, I also seem to miss the step on how to do this.

 A suggestion on the implementation section, it would be nice to have a graphical block diagram that shows the steps and inputs required for KnetMaps to be installed in an end-user's system. A statement reporting on installation and functionality of the system under 3 dominant OSs would be appreciated (Linux, Unix/MacOS, MSWindows?), as upfront knowledge for users who wish to install this in their own systems.

I have read this submission. I believe that I have an appropriate level of expertise to confirm that it is of an acceptable scientific standard.

# Peer review: Referee response for version 1

**DOI:** 10.5256/f1000research.18148.r42478

**Published:** 2019-01-25

**Authors:** Nicholas J. Provart

**Affiliations:** 1 Department of Cell and Systems Biology, Centre for the Analysis of Genome Evolution and Function, University of Toronto, Toronto, Canada

In general the concept of the KnetMaps BioJS component is very welcome. I like the functionality that the authors have developed (at least as described in the paper), but I think they could take it up a notch to use additional “visualization techniques to prevent information overload”, especially taking advantage of Cytoscape.js’s ability to group nodes via the compound node feature – this way, different types of nodes could be grouped (which might be useful for larger networks where things might become quite overloaded). Nodes and edges might receive some additional treatment. 

 The authors write: “Developers can customize the stylesheet to replace the supported static cytoscapeJS shapes (such as triangle, round-rectangle, ellipse, pentagon and star) with images.” It would be nice to actually include a set of image-based icons for the different nodes in the KnetMaps release. Given the multiple types of nodes possible, perhaps a node icon that looks like a page with a few lines on it could symbolize publications, a node icon with an stylized alpha helix could represent the protein etc. I’d also explore using different kinds of edges (straight, curved, dashed) instead of a just a colour scheme.

 Sample.json did not seem to be in NPM (as described in the code in the article)? I tried getting KnetMaps to work within Codepen.io and unkpg.com/knetmaps/ but wasn’t quite able to. It would be nice to have this set up so readers could try out the package simply within a browser (by making the “pen” public and sharing the link in the F1000 article). It was almost working, except the JSON file (e.g. Arabidopsis flower network) couldn’t be loaded because of a MIME type error and even when I included the JSON object in the script it wasn’t working (see code here). Another reason for providing a Codepen link is that there is no preview on the BioJS site.

I have read this submission. I believe that I have an appropriate level of expertise to confirm that it is of an acceptable scientific standard, however I have significant reservations, as outlined above.

# Peer review: Referee response for version 1

**DOI:** 10.5256/f1000research.18148.r42477

**Published:** 2019-01-24

**Authors:** Chia-Yi Cheng

**Affiliations:** 1 Center for Genomics and Systems Biology, New York University, New York, NY, USA

This article describes a package KnetMaps that provides a JavaScript-based tool to visualize content-rich biological data. KnetMaps is suited for a website with heterogeneous biological information by integrating results in a network format. The examples listed on the demo page ( http://knetminer.rothamsted.ac.uk/KnetMaps/) allows users to taste the flavor of KnetMaps. The interface is intuitive and straightforward. Below are suggestions to further enhance the clarity and completeness of the user experience:

1. It would be beneficial for both the developers and end users if the author could provide a step-by-step guide to reproduce one example dataset as on the demo page.
2. The ‘interactive legend’ feature allows users to add an additional layer to the default network. It is not completely clear to me: i) how was the visible/invisible edges/nodes set in the first place? Ii) is it possible to remove the once added information? For example, clicking on a ‘Domain’ symbol in the legend will add ‘Domains’ linked to visible nodes. A user may review and find the information not needed and want to hide those edges. I may have missed it but I did not find a way to cherry pick the symbols once the associated edges displayed.  
3. The PNG is working on the demo page ( http://knetminer.rothamsted.ac.uk/KnetMaps/) but not the use case pages ( http://knetminer.rothamsted.ac.uk/Zea_mays/) ( http://daisychain.appliedbioinformatics.com.au/). A blank window popped up when I hit the PNG icon.

I have read this submission. I believe that I have an appropriate level of expertise to confirm that it is of an acceptable scientific standard.

# Peer review: Referee response for version 1

**DOI:** 10.5256/f1000research.18148.r39702

**Published:** 2018-11-01

**Authors:** Ian Dunham, Gareth Peat

**Affiliations:** 1 Open Targets, EMBL-EBI, Cambridge, UK

This is a useful article and software package to help apply cytoscape.js to heterogeneous knowledge graphs. KnetMaps.js adds several features surrounding the cytoscape.js graph visualization, such as the legend, information panel and download functionality. Following the setup guide to run the sample application locally was fairly straightforward.

 The software is available online to test at both http://knetminer.rothamsted.ac.uk/KnetMaps/ and http://daisychain.appliedbioinformatics.com.au/. Developers can obtain the source through npm and biojs, where the package is named knetmaps.

 KnetMaps.js requires that input data be in a specific format and there are examples of this in the setup guide. The format is easy to understand, but combines data and styling information per node or edge. Separation of these concerns would add clarity and conciseness.

 A user wishing to display heterogeneous data, i.e. data containing different node and edge types, might find KnetMaps.js could save them development time. Several features that one might want to add on top of cytoscape.js are provided out of the box, such as the interactive legend, node/edge information panel, PNG/JSON export functionality, filtering and a variety of layout algorithms. However, for any substantial deviation from the UI design provided, a developer might be tempted to start with cytoscape.js directly, which has many online examples and is well documented.

 Minor revisions:

- Software

1. PNG export. This functionality does not currently include the interactive legend, which would be useful to explain the static image.
2. File extensions. The sample files have misleading file extensions. For example, ara2.json, which has JavaScript content, should really be a .js file.
3. Automated build step. The running of gulp optimize could be done prior to publishing on npm. This would make it even easier for an end user to embed the necessary .js and .css files.
4. Descriptions for example datasets. There are several example datasets available at http://knetminer.rothamsted.ac.uk/KnetMaps/. It would be helpful to provide a brief description of where the datasets came from and what they contain.

- Manuscript

1. Use of bower. I did not need to use bower to set up the sample application and would suggest removing the reference in the paper.

Longer term suggestions for improvement:

1. Add some unit tests. This would also double as documentation for developers.
2. Use npm to install third party libraries. Several libraries, such as cytoscape, cytoscape-cose-bilkent are not installed using npm. Upgrading them would be more straightforward if the package manager were used.

We have read this submission. We believe that we have an appropriate level of expertise to confirm that it is of an acceptable scientific standard.
